# Prognosis and Concurrent Genomic Alterations in Patients With Advanced NSCLC Harboring MET Amplification or MET Exon 14 Skipping Mutation Treated With MET Inhibitor: A Retrospective Study

**DOI:** 10.3389/fonc.2021.649766

**Published:** 2021-06-24

**Authors:** Li Liu, Farhin Shaheed Kalyani, Haiyan Yang, Chunhua Zhou, Yi Xiong, Songlin Zhu, Nong Yang, Jingjing Qu

**Affiliations:** ^1^ Department of Lung Cancer and Gastroenterology, Hunan Cancer Hospital, Affiliated Tumor Hospital of Xiangya Medical School of Central South University, Changsha, China; ^2^ Department of Respiratory Disease, The First Affiliated Hospital, School of Medicine, Zhejiang University, Zhejiang, China; ^3^ Department of Clinical Pharmaceutical Research Institution, Hunan Cancer Hospital, Affiliated Tumor Hospital of Xiangya Medical School of Central South University, Changsha, China

**Keywords:** concurrent genomic alterations, MET inhibitor, MET amplification, MET exon 14 skipping mutation, non-small cell lung cancer

## Abstract

**Background:**

MET amplification or METex14 skipping mutations are uncommon oncogenic events in NSCLC patients. Clinicopathological characteristics, concurrent gene alterations, and prognosis of MET TKIs in these patients are yet to be elucidated.

**Methods:**

We retrospectively analyzed the genomic profiles of 43 MET amplifications or 31 METex14 skipping mutations in NSCLC patients with no previous treatment with EGFR TKIs. Survival outcomes were analyzed in evaluable patients receiving MET TKI treatment: MET amplification cohort (n = 29) and METex14 skipping mutation cohort (n = 29).

**Results:**

Among evaluable patients, a shorter PFS was observed in the MET amplification cohort than in the METex14 skipping mutation cohort (7.0 months *vs.* 11.0 months, P = 0.043). Concurrent mutations in both cohorts resulted in a statistically significant shorter PFS (MET amplification: 3.5 months *versus* 8.0 months, P = 0.038, METex14 skipping mutation: 7.0 *versus* NR months, P = 0.022). However, a statistically significant OS (17.0 months *versus* 20.0 months, P = 0.044) was only observed in the MET amplification cohort. TP53, the most common concurrent mutation in both cohorts, was associated with worse survival outcomes as compared to the wild type. The MET amplification cohort with a concurrent PIK3CA mutation exhibited primary resistance to MET TKIs and showed disease progression (80%).

**Conclusion:**

MET TKIs could be a better treatment option for patients with METex14 skipping mutations. Concurrent mutations may deteriorate the PFS of MET TKIs in NSCLC patients with MET amplification or METex14 skipping mutations. PIK3CA mutations may confer primary resistance to MET TKIs in patients with MET amplification.

## Introduction

Non-small cell lung cancer (NSCLC) is the leading cause of tumor-related deaths worldwide. Molecular heterogeneity, proliferation, and metastasis in NSCLC have been associated with various driver mutations in the epidermal growth factor receptor (EGFR), anaplastic lymphoma kinase (ALK), proto-oncogene tyrosine-kinase protein (ROS1), and mesenchymal epithelial transition factor receptor (MET) (1, 2). MET, a receptor tyrosine kinase (RTK) located on chromosome 7q21-31, encodes a heterodimeric transmembrane RTK, which is activated upon binding of hepatocyte growth factors (HGF). This process results in the downstream signaling of phosphatidylinositol 3-kinase (PI3K) and mitogen-activated protein kinase (MAPK) pathways, subsequently leading to tumor proliferation, progression, and metastasis (3–6). Several mechanisms, including gene amplification, overexpression, mutations, and fusion, can cause deregulated MET signaling (7, 8). MET amplification in NSCLC has been identified as a common mechanism of resistance (2–8%) to EGFR tyrosine kinase inhibitors (TKIs) in patients with no prior drug exposure (8). Moreover, MET amplification is also a potential resistance mechanism to the third EGFR-TKI, osimertinib (9, 10).

MET exon 14 (METex14) skipping mutations have recently been found to be oncogenic, seen in approximately 3 to 4% of NSCLC cases, and are associated with MET amplification and overexpression (11–13). Response to MET TKIs, such as crizotinib and cabozantinib, have been reported in NSCLC patients with MET amplification and METex14 skipping mutations. AcSé phase II trial showed a 16% overall response rate (ORR) in the MET amplification cohort (25 patients) treated with crizotinib (14). In the PROFILE 1001 study, 28 patients with METex14 skipping mutations receiving crizotinib had an ORR of 39% with a median progression-free survival (mPFS) of 8 months (12, 15, 16). Furthermore, preliminary data from a case report of primary resistance to crizotinib have been reported in patients with METex14 skipping (17). The underlying mechanism of primary resistance to MET inhibitors is yet to be elucidated.

Fluorescence *in situ* hybridization (FISH) was the most commonly used method in detecting chromosomal abnormalities. In recent years, next-generation sequencing (NGS) has become a more popular method and is being integrated into routine clinical oncology practice. Targeted NGS allows the simultaneous integration of multiple genes and provides a comprehensive mutation profile of not only actionable mutations, but also other gene mutations associated with cancer development. Concurrent driver genes with sensitizing mutations have been shown to affect the survival outcomes of targeted therapies.

Our study aimed to elucidate the comprehensive mutation profile of patients with MET amplification or METex14 skipping mutations in NSCLC receiving MET TKI therapy by analyzing molecular factors that could contribute to their prognosis. In fact, no retrospective study has previously evaluated the efficacy of MET TKIs in the treatment of patients with *de novo* MET amplifications and METex14 skipping mutations.

Herein, we describe the survival characteristics by analyzing mutated genes and underlying primary resistance factors with MET amplification and METex14 skipping mutations.

## Materials and Methods

### Population and Respondents

Patients diagnosed with NSCLC with MET amplification (n = 43) or METex14 skipping mutations (n = 31) from Hunan Cancer Hospital between March 2015 and October 2020 were retrospectively analyzed. Baseline MET amplification or METex14 skipping mutation status was assessed using blood or tissue samples obtained *via* needle biopsy of lung lesions or lymph nodes. Samples were sent to Buring Rock Biotech for molecular genotyping using NGS (56 gene or 168 gene panel). The following patients were excluded: (1) patients previously treated with other EGFR TKIs before MET detection and (2) patients with evidence of small cell lung cancer (SCLC) metastasis. This study was approved by the Institutional Review Board of Hunan Cancer Hospital. All patients provided written informed consent for the use of their data in this study.

### Treatment Procedures

MET TKIs, including 250 mg crizotinib twice daily, 200 mg bozitinib twice daily, or 600 mg volitinib once daily, were prescribed to the MET amplification or METex14 skipping mutation cohorts. Treatment was discontinued if unacceptable toxicity, disease progression or death, patient refusal, or treatment withdrawal for any other reason, including pregnancy, were noted. Response was measured using enhanced computed tomography (CT) scans in accordance with the Response Evaluation Criteria in Solid Tumors (RECIST) version 1.1 (18). The objective response rate (ORR) was defined as the proportion of patients achieving complete response (CR) or partial response (PR). Disease control rate (DCR) was defined as the proportion of patients achieving CR, PR, and stable disease (SD). Treatment-related toxicity was evaluated according to the Common Terminology Criteria for Adverse Events version 4.03. Progression-free survival (PFS) was defined as the period from treatment initiation to discontinuation due to radiologically confirmed disease progression, intolerable toxicity, or death. Overall survival (OS) was defined as the period from the date of treatment initiation with MET TKIs until death or the day of the last follow-up.

### Preparation of Plasma-Circulating, Cell-Free DNA (cfDNA), and Tissue DNA

Circulating cfDNA was extracted from plasma samples according to the manufacturer’s instructions using a QIAamp Circulating Nucleic Acid Kit (Qiagen, Hilden, Germany). Similarly, tissue DNA from formalin-fixed, paraffin-embedded (FFPE) cell blocks of tumor biopsy or other cytologic samples were extracted using the QIAamp DNA FFPE tissue kit (Qiagen, Hilden, Germany). A Qubit 2.0 Fluorometer with dsDNA HS Assay Kit (Life Technologies, CA, USA) was used to quantify cfDNA and tissue DNA concentration.

### NGS Library Construction

NGS library construction was performed using optimized protocols developed by Burning Rock Biotech (19). Tissue DNA was ultrasonicated using a Covaris M220 (Covaris, MA, USA). Fragments of 170 bp for plasma cfDNA and of 200–400 bp for sheared tissue DNA were purified with magnetic beads using an Agencourt AMPure XP kit (Beckman Coulter, CA, USA). Purified fragments were then hybridized with capture probes, hybrid-selected with magnetic beads, and amplified using PCR. The quality and size of the fragments were assessed using an Agilent high-sensitivity DNA assay kit and a Bioanalyzer 2100 (Agilent, CA, USA). Sequencing of the indexed samples was performed using a NextSeq500 (Illumina, CA, USA) with paired-end reads at a target sequencing depth of 10,000× for plasma samples and 1,000× for tissue samples.

### Sequence Data Analysis

NGS sequence data were analyzed using an optimized pipeline developed by Burning Rock Biotech (19). Sequence mapping to the reference human genome (hg19) was performed using GATK (version 3.2). Moreover, VarScan (version 2.4.3) was used for local alignment optimization, variant calling, and annotation. Genomic DNA profiling was conducted *via* capture-based targeted sequencing using commercial gene panels. These panels, including either the 56 gene or 168 gene panel (Burning Rock Biotech, Guangzhou, China), interrogated all exons and critical introns of classic lung cancer oncogenes (19). Moreover, these panels also facilitated the detection of various mutation types, including single nucleotide mutations (SNMs), copy number variations (CNVs), and structural variations. CNV was defined as the coverage data of the gene region that were quantitatively and statistically significant from its reference control. Coverage depth data were first corrected for sequencing bias due to GC content and target probe density. The average coverage of all capture regions was calculated as an internal control, which was utilized to normalize the coverage of different samples to comparable scales. The coverage of MET with copy number gains was significantly greater than that of the internal control. The cut-off for identifying gene copy number (GCN) deletion was set at a GCN of 1.5, and amplification at a GCN of 2.25 (20). The difference in the adjusted coverage depth for each gene between the samples and the reference was evaluated using the t-test method. Using the GCN≥4 restriction, MET amplification was divided into three intensity levels: (1) No amplification: MET-Ratio<1.8; (2) Low amplification: 1.8≤MET-Ratio ≤ 2.2; and (3) Intermediate amplification: 2.2<MET-Ratio<5; High: MET-Ratio≥5 (21).

### Statistical Analysis

Survival analysis was performed for each group using the Kaplan-Meier method with log-rank statistics. Either the Fisher’s exact test, Chi-square test for trend or the paired two-tailed Student’s t-test was used to calculate the statistical differences between the groups. All statistical analyses were performed using the GraphPad Prism 8 software. Statistical significance was defined as p < 0.05.

## Results

### Patient Characteristics

This study included 43 patients in the MET amplification cohort and 31 patients in the METex14 skipping mutation cohort admitted at Hunan Cancer Hospital from March 2015 to October 2020. Tissue or blood samples were sent to Burning Rock Biotech for sequencing prior to MET TKI therapy. In the MET amplification cohort, the median age was 56 years, ranging from to 37 to 76 years. On the other hand, the METex14 skipping mutation cohort had a median age of 61 years, ranging from 41 to 81 years. Regarding NSCLC histology, adenocarcinoma was most often detected in both cohorts: MET amplification cohort, 83.7% (36/43); METex14 skipping mutation cohort, 83.9% (26/31). Squamous cell carcinoma was detected in the remaining patients: MET amplification cohort, 16.3% (7/43); METex14 skipping mutation cohort, 16.1% (5/31).

The disease stage upon initial diagnosis was I-IIIa in 14.0% (6/43), IIIb-IIIc in 2.3% (1/43), and IV in 83.7% (36/43) in the MET amplification cohort. On the other hand, all patients in the METex14 cohort were in the advanced stage, with 90.3% (28/31) of patients at stage IV and the remaining 9.7% (3/31) at stage IIIb-IIIc. In the MET amplification cohort, 67.4% (29/43) of the patients received MET TKI treatment (25/29 with crizotinib, 4/29 with bozitinib). The remaining 32.6% (14/43) did not receive MET TKI therapy because of early-stage post-surgery (6/14) or refusal of treatment (8/14). Of the 29 patients receiving therapy, 82.8% (24/29) received it as a first-line regimen, while the remaining 17.2% (5/29) received it as a second-line treatment. In the METex14 skipping mutation cohort, 93.5% (29/31) of patients received MET TKIs (22/29 with crizotinib, 5/29 with bozitinib, 2/29 with volitinib). The remaining 6.5% (2/31) of the patients did not receive any therapy due to refusal of treatment. Of the 29 patients receiving therapy, 86.2% (25/29) received it as a first-line regimen, while the remaining 13.8% (4/29) received it as a second-line treatment. In addition, 60.5% (26/43) of the MET amplification cohort and 64.5% (20/31) of the METex14 cohort had concurrent gene mutations. Both cohorts were independent of sex, age, smoking status, and clinical stage. The clinicopathologic characteristics of the two cohorts are summarized in **Table 1**.

**Table 1:** Summary of baseline clinical characteristics of patients identified with MET amplification and MET ex14 skipping mutation.

Characteristic	MET amplification (n = 43) n (%)	METex14 skipping mutation (n = 31) n (%)	P
**Gender**			0.79
Male	32 (74.4%)	22 (71.0%)	
Female	11 (25.6%)	9 (29.0%)	
**Median age (range)**	56 (37–76)	61 (41–81)	0.32
**Smoking History**			0.34
Non-smoker	15 (34.9%)	15 (48.4%)	
Smoker	28 (65.1%)	16 (51.6%)	
**Histologic type**			>0.99
Adenocarcinoma	36 (83.7%)	26 (83.9%)	
Squamous cell carcinoma	7 (16.3%)	5 (16.1%)	
**Stage**			0.13
I-IIIa	6 (14.0%)	0 (0.0%)	
IIIb-IIIc	1 (2.3%)	3 (9.7%)	
IV	36 (83.7%)	28 (90.3%)	
**Brain metastasis**			>0.99
Yes	7 (16.3%)	5 (16.1%)	
No	36 (83.7%)	26 (83.9%)	
**MET-TKIs therapy**			0.052
Crizotinib	25 (58.1%)	22 (71.0%)	
Volitinib	0 (0.0%)	2 (6.5%)	
Bozitinib	4 (9.3%)	5 (16.0%)	
Without MET-TKIs therapy	14 (32.6%)	2 (6.5%)	
**Line of MET-TKI treatment**			0.009
First-line	24 (55.8%)	25 (80.6%)	
Subsequent-line	5 (11.6%)	4 (12.9%)	
Without MET-TKIs therapy	14 (32.6%)	2 (6.5%)	
**Concurrent mutation**			0.81
With	26 (60.5%)	20 (64.5%)	
Without	17 (39.5%)	11 (35.5%)	

### Clinical Outcomes of Patients Harboring MET Amplification or METex14 Skipping Mutation Treated With MET TKIs

**Figure 1:**
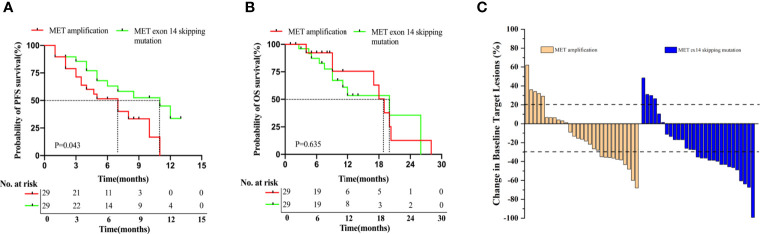
Patients with METex14 skipping mutation treated with MET TKIs have significantly longer PFS. Kaplan-Meier estimation of PFS **(A)**, OS **(B)**, and ORR **(C)** of MET TKI treated patients with MET amplification and METex14 skipping mutation. PFS and OS are expressed in months. Dotted line indicates the median survival.

In this study, 67.4% (29/43) and 93.5% (29/31) of patients from the MET amplification or METex14 skipping mutation cohort, respectively, who received MET TKI treatment were included in the analysis. Log-rank analysis showed a statistically significant shorter PFS in the MET amplification cohort than in the METex14 skipping mutation cohort (7.0 months *vs.* 11.0 months, P = 0.043, **Figure 1A**). However, results showed a statistically insignificant OS in patients with MET amplification as compared to those with METex14 skipping mutation (19.0 months *vs.* 20.0 months, P = 0.635; **Figure 1B**). In the MET amplification group who received MET TKIs, patients with intermediate MET amplification showed insignificant PFS (5.0 months *vs.* 8.0 months, P = 0.556) and OS (17.0 months *vs.* 18.0 months, P = 0.923) as compared to those with high MET amplification (**Supplementary Figures 1A, B**).

Among the 29 patients who received MET TKIs in the MET amplification group, 34.5% (10/29) achieved PR, 48.3% (14/29) achieved SD, and 17.2% (5/29) achieved PD, resulting in an ORR of 34.5% and a DCR of 82.8%. Of the 29 patients who received MET TKIs in the METex14 skipping mutation group, 51.7% (15/29) achieved PR, 34.5% (10/29) achieved SD, and 13.8% (4/29) achieved PD, resulting in an ORR of 51.7% and DCR of 86.2%(**Figure 1C**). Overall, these data suggested that patients with MET14 skipping mutations had a significantly longer PFS than patients with MET amplification. Meanwhile, the ORR was higher in the METex14 skipping mutation cohort than in the MET amplification cohort.

### Mutation Profiles of the Cohorts

**Figure 2:**
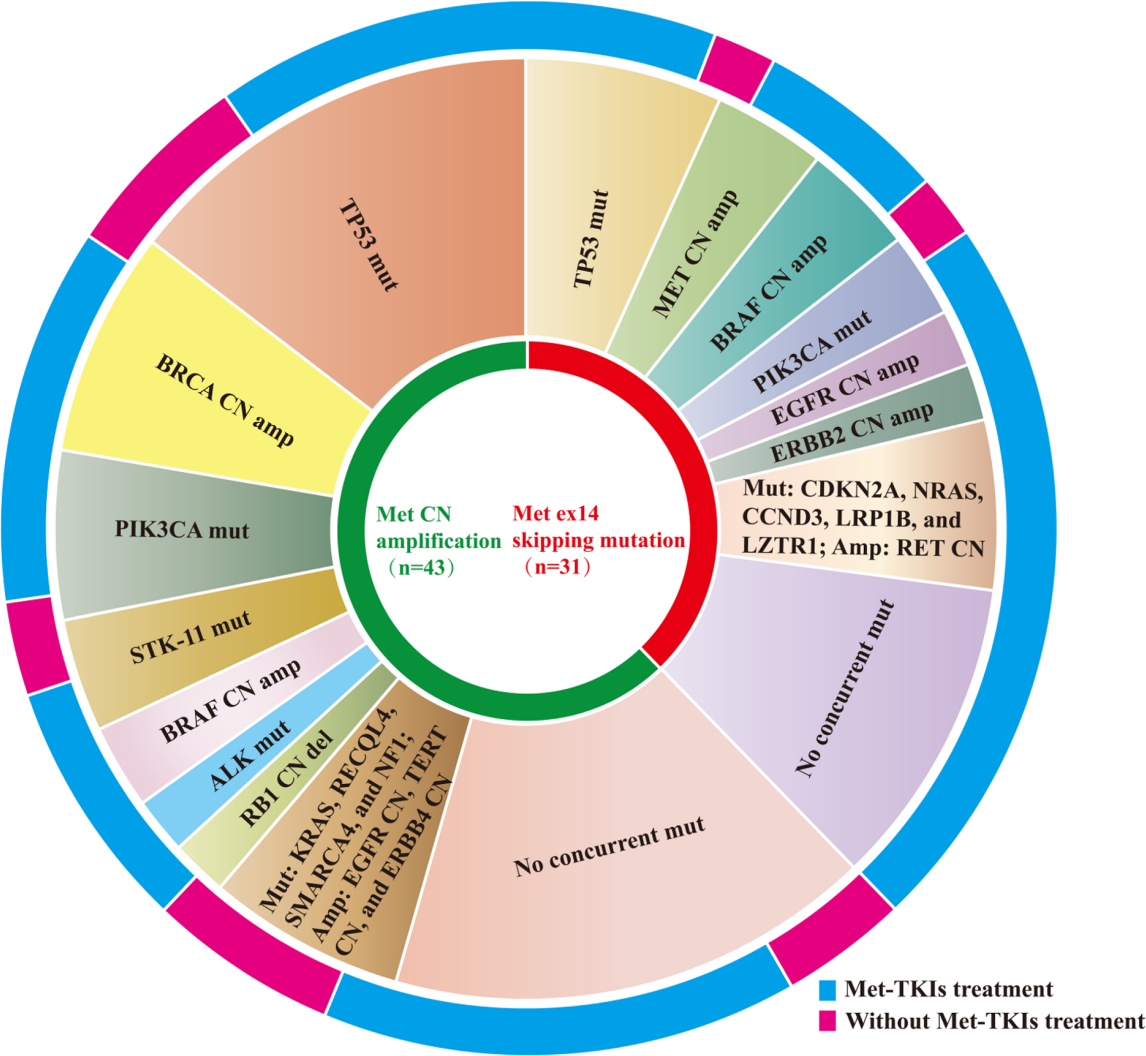
Distribution of concurrent mutation detected in MET amplification and METex14 skipping mutation cohorts.

Target gene sequencing using a panel of 56 and 168 cancer-related genes was performed on tissue samples collected from patients to elucidate the baseline comprehensive mutation status prior to initial MET TKI therapy. Results showed that 60.5% (26/43) of the MET amplification cohort and 64.5% (20/31) of the METex14 skipping mutation cohort had other concurrent mutations. In the MET amplification cohort, concurrent TP53 mutations were the most common, affecting 34.9% (15/43) of the patients. In addition, other concurrent mutations, including BRCA CN amplification (n = 8), PIK3CA mutation (n = 6), STK-11 mutation (n = 4), BRAF CN amplification (n = 3), ALK missense mutation (n = 2), RB1 CN deletions (n = 2), RECQL4 mutation (n = 1), KRAS mutation (n = 1), EGFR CN amplification (n = 1), TERT CN amplification (n = 1), SMARCA4 mutation (n = 1), NF1 mutation (n = 1), and ERBB4 CN amplification (n = 1) were also detected. Regardless, 39.5% (17/43) of the patients in the MET amplification cohort did not harbor any concurrent gene mutations. Moreover, TP53 mutation was also observed in the METex14 skipping mutation cohort, accounting for 22.6% (7/31) of the patients. Other gene mutations detected in the METex14 cohort included MET CN amplification (n = 4), BRAF CN amplification (n = 4), PIK3CA mutation (n = 3), EGFR CN amplification (n = 2), ERBB2 CN amplification (n = 2), RET CN amplification (n = 1), CDKN2A mutation (n = 1), NRAS mutation (n = 1), CCND3 mutation (n = 1), LRP1B mutation (n = 1), and LZTR1 mutation (n = 1). Regardless, 35.5% (11/31) of the patients did not harbor any concurrent mutations (**Figure 2**). Concurrent mutations observed in both cohorts are summarized in **Tables S1, S2**.

### Influence of Concurrent Mutations to Survival Outcomes With MET TKI Therapy

In this study, we analyzed the presence or absence of concurrent mutations associated with survival outcomes to further explore other molecular factors that may influence patient prognosis. As discussed above, 26/43 patients in the MET amplification cohort were found to have concurrent gene mutations. Of these, 16 received treatment with MET TKIs (61.5%, 16/26). Of the remaining 17 patients without concurrent gene mutations in the MET amplification cohort, 13 received MET TKI therapy (76.5%, 13/17).

**Figure 3:**
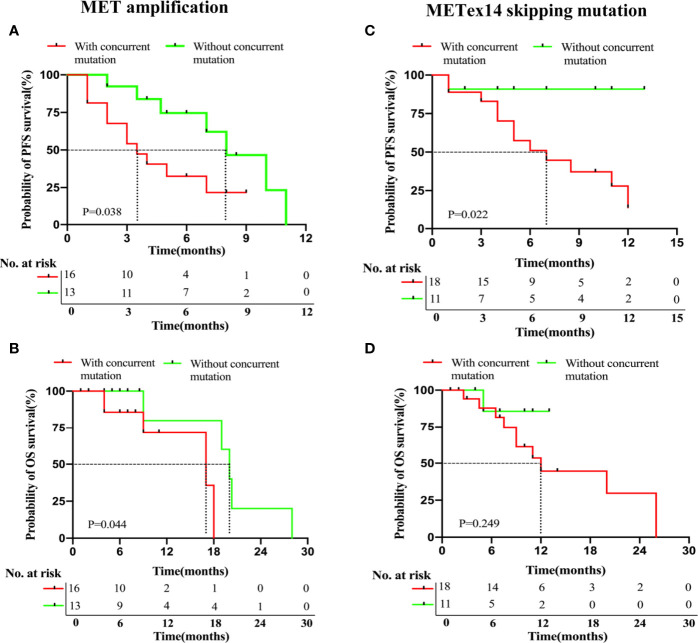
NSCLC patients with MET amplification and METex14 skipping mutation harboring concurrent deleterious mutations have significantly shorter PFS with MET TKI therapy. Kaplan-Meier analyses for PFS **(A, C)** and OS **(B, D)** of patients based on the presence or absence of concurrent mutations in MET amplification and MET ex14 skipping mutation cohorts.

We compared the PFS and OS of the patients. In the MET amplification cohort who received MET TKIs, we compared patients with and without concurrent mutations (n = 16 and 13, respectively). Statistically significant shorter PFS (3.5 months *vs.* 8.0 months, P = 0.038, **Figure 3A**) and OS (17.0 months *vs.* 20.0 months, P = 0.044, **Figure 3B**) were both noted in patients with a concurrent mutation as compared to those without, respectively. We similarly compared those with and without concurrent mutations in the METex14 cohort who received MET TKI therapy (n = 18, n = 11, respectively). A statistically significant shorter PFS (7.0 *vs.* NR months, P = 0.022, **Figure 3C**) but a statistically insignificant OS (12.0 *versus* NR months, P = 0.249; **Figure 3D**) were noted in patients with a concurrent mutation as compared to those without, respectively.

**Figure 4:**
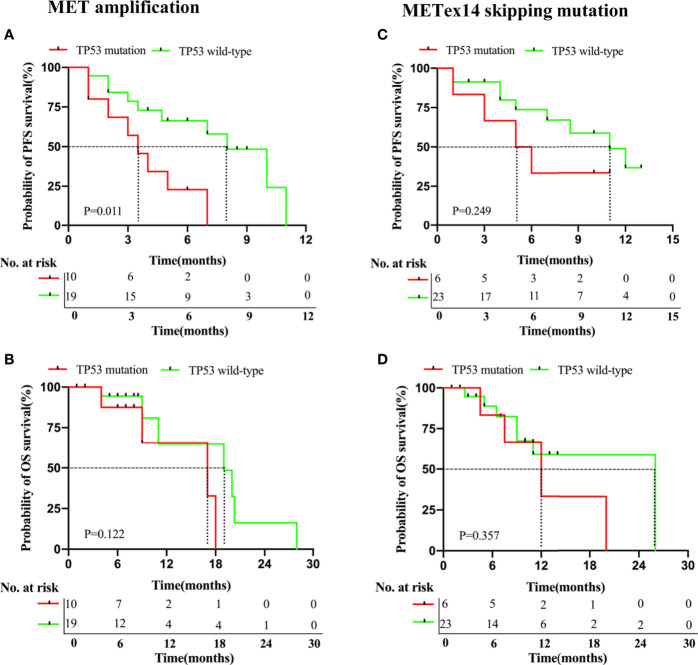
NSCLC patients with MET amplification and METex14 skipping mutation harboring TP53 mutations have deleterious survival outcomes with MET TKI therapy. Kaplan-Meier curves of PFS **(A, C)** and OS **(B, D)** of patients treated with MET TKIs based on presence or absence of concurrent TP53 mutation in MET amplification and METex14 skipping mutation cohorts.

We studied the effects of other molecular factors on survival outcomes. We found that several studies have shown an association between concurrent TP53 mutations and poorer survival outcomes with TKI therapy. Since TP53 was the most common concurrent mutation in both cohorts, we investigated its influence on the clinical outcomes of each cohort. Among the patients of the MET amplification cohort who were treated with MET TKIs (29/43), those with a TP53 mutation (n = 10) had a statistically significant shorter PFS (3.5 *vs.* 8.0 months, P = 0.011, **Figure 4A**) but an insignificant OS (17.0 months *vs.* 19.0 months, P = 0.122, **Figure 4B**), as compared to those with a wild-type TP53 (n = 19).

Among the patients of the METex14 skipping mutation cohort who received MET TKI therapy (29/31), those with a TP53 mutation (n = 6) had an insignificantly shorter PFS (5.5 months *vs.* 11.0 months, P = 0.249, **Figure 4C**) and OS (12.0 *vs.* 26.0 months, P = 0.357; **Figure 4D**), as compared to those with a wild-type T53 (n = 23).

In summary, these data suggested that a concurrent gene mutation can influence the survival outcomes of patients who received MET TKI therapy in both cohorts, particularly in the MET amplification cohort with a concurrent TP53 mutation.

### Patients With a PIK3CA Mutation in the MET Amplification Cohort Displayed Primary Resistance to MET TKIs

PIK3CA mutations have been found to be involved in TKI resistance in several cancer models. Therefore, we focused on analyzing PIK3CA mutations in both cohorts. NGS testing revealed PIK3CA mutations in 14.0% (6/43) of the MET amplification cohort, of which 5/6 have undergone MET TKI therapy (four patients achieved PD and one patient achieved PR) while 1/6 received chemotherapy. In this cohort, those with a PIK3CA mutation who received MET TKI therapy (n = 5) were found to have a statistically significant shorter PFS (1.0 month *vs.* 7.0 months, P = 0.004, **Supplemental Figure 2A**) but a statistically insignificant shorter OS (11.0 months *vs.* 19.0 months, P = 0.058, **Supplemental Figure 2B**), as compared to those who received MET TKI therapy but without a PIK3CA mutation (n = 24).

In the METex14 cohort, 9.7% (3/31) of the patients were found to have PI3KCA mutations, of which 2/3 received therapy with MET TKIs (one patient achieved PD and one patient achieved PR), while one patient refused any therapy. Due to the small cohort size, the relationship between survival outcomes and PIK3CA mutation in the METex14 skipping mutation cohort could not be analyzed.

**Figure 5:**
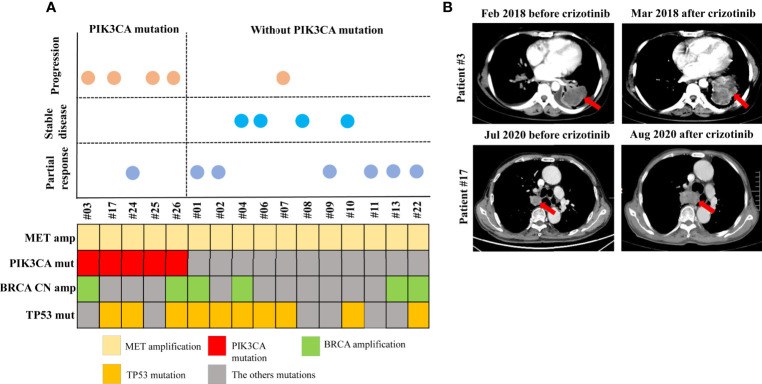
Patients with a PIK3CA mutation in MET amplification cohort who received MET TKIs showed progressive disease. Schematic presentation of patients according to the response to MET TKI treatment, and to the presence or absence of a PIK3CA mutation **(A)**. Computed tomography scans of patient #3 and patient #17 showing disease progression after 1 month of MET TKI treatment **(B)**.

Four patients from the MET amplification cohort who had concurrent PIK3CA mutations and had received MET TKIs showed progressive disease at the first radiologic assessment (**Figures 5A, B**). Details of the clinical course of two of the patients are as follows. Patient #3, with MET amplification, BRCA amplification, and PIK3CA mutation, was a 51-year-old woman diagnosed with stage IV NSCLC with liver metastasis. She received first-line crizotinib, but progression was observed after 1 month of treatment due to rapid developments of the lung mass and liver metastases. Furthermore, the patient received additional chemotherapy, which was deemed ineffective. She died 11 months after diagnosis (**Figure 5B**). Patient #17, with MET amplification, PIK3CA mutation, and TP53 mutation, was a 69-year-old male and a former smoker diagnosed with stage IV adenocarcinoma. He received first-line crizotinib, which was discontinued upon the first radiologic assessment due to primary lesion progression (**Figure 5B**). The patient was found to have high PDL-1 expression and was therefore started on an anti-PD-1 agent. The patient is currently undergoing follow-up.

## Discussion

MET is emerging as a clinically relevant biomarker for predicting treatment response with MET inhibitors. MET amplification or METex14 skipping mutation has been implicated as an oncogenic driver in NSCLC and has been proposed as a potential therapeutic target. Most studies regarding MET amplification and METex14 skipping mutation have been carried out in Western populations (5, 22, 23), and the prevalence of these types of MET alterations in the Chinese population has yet to be elucidated. In addition, no studies have compared the clinical characteristics and survival outcomes between patients with MET amplification and METex14 skipping mutations who received first-line therapy with MET TKIs. To the best of our knowledge, this is the first retrospective study to compare clinical characteristics, survival outcomes, and concurrent genomic mutations in NSCLC in patients of Chinese ethnicity harboring MET amplification or METex14 skipping mutation. We also investigated potential molecular markers for predicting the survival outcomes of patients with MET amplification and METex14 skipping mutations.

NSCLC with MET amplification is a new potential target, particularly in patients with a history of smoking. A multiple-cohort, phase II study performed in patients with MET amplification showed an ORR of 40% in those who received capmatinib (22). A retrospective study investigating the efficacy of crizotinib in Chinese NSCLC patients with *de novo* MET amplification showed an mPFS of 6.5 months (24). Our results revealed an mPFS of 7.0 months and an ORR of 34.5% in the MET amplification cohort who received treatment with MET TKIs. Despite the small sample size, our findings were consistent with those of other studies showing the efficacy of MET TKIs in NSCLC patients with MET amplification. In our study, patients with intermediate MET amplification showed insignificant PFS and OS as compared to those with high MET amplification who received MET TKIs. However, a longer PFS was observed with high-level amplification than that with intermediate-level amplification. This is supported by a previous study showing that high-level amplification is more selective for treatment response as compared to low-level amplification (25, 26). Furthermore, several studies focusing on METex14 skipping mutations as target alterations in NSCLC have also been published (27–29). Paik et al. reported eight patients with the METex14 skipping mutation, four of whom received crizotinib or cabozantinib and achieved PR (12). The expansion cohort of the PROFILE 1001 clinical trial which included 65 patients with NSCLC harboring the METex14 skipping mutation showed an ORR of 32% with crizotinib, with three patients achieving CR and 18 patients achieving PR. The median PFS and OS of these patients were 7.3 months and 20.5 months, respectively (30). Another study retrospectively analyzed the survival outcomes in patients with METex14 skipping mutation. The PFS and OS of patients receiving crizotinib were 8.0 months and 11.3 months, respectively (31). In addition, the prevalence of 1.1% for MET-ex14 alterations is more likely to be detected in older patients. In patients harboring MET-ex14 alterations, longer PFS were observed with crizotinib than with chemotherapy (31).

In our METex14 skipping mutation cohort, the median PFS was 11.0 months, OS was 20.0 months, and ORR was 51.7%, similar to previous studies. Interestingly, our study found that among patients who received treatment with MET TKIs, those in the MET amplification cohort showed a statistically significant PFS, as compared to those in the METex14 skipping mutation cohort (P = 0.043). Although several previous studies have reported the survival outcome in NSCLC patients with MET amplification and METex14 skipping mutation patients, no clinical study has directly compared the survival outcomes of these two genetic alterations. Our study is the first retrospective analysis of various outcomes in patients with NSCLC, with either MET amplification or METex14 skipping mutation, receiving treatment with MET TKIs. Our results suggest that MET TKI therapy in NSCLC patients with METex14 skipping mutations is more efficacious than MET amplification.

Generally, patients with MET amplification or METex14 skipping mutation have a variable response to the same treatment, suggesting the presence of another factor contributing to molecular heterogeneity. Therefore, we further analyzed the comprehensive mutation profile of the patients in our cohort to identify whether a concurrent mutation would influence the clinical outcomes of these patients. Several studies have shown that concurrent mutations in lung cancer are associated with worse survival outcomes and resistance to TKIs (32, 33). Moreover, another study showed that patients harboring concurrent mutations in addition to METex14 skipping mutations were resistant to MET TKIs (34).

In our study, 60.5% (26/43) of patients with MET amplification and 64.5% (20/31) of patients with METex14 skipping mutations harbored a concurrent gene mutation. Of these patients, those who received treatment with MET TKIs were found to have a significantly shorter PFS, as compared to those without a concurrent mutation. Studies have also shown that a concurrent TP53 mutation is associated with reduced responsiveness to TKIs and a relatively worse prognosis (35–38). In our study, TP53 was the most common concurrent mutation, detected in 34.9% of the MET amplification cohort and 22.6% of the METex14 skipping mutation cohort. Among those in the MET amplification cohort who received MET TKI treatment, patients with concurrent TP53 mutation resulted in a shorter PFS (P = 0.011) than in TP53 wild-type patients, indicating TP53 as a potential molecular marker for predicting survival outcomes in patients with MET amplification. However, among those in the METex14 skipping mutation cohort who received TKI treatment, we found that patients with TP53 mutation had a significantly shorter PFS (5.5 *versus* 11.0 months, P = 0.357) and OS (12.0 *versus* 26.0 months, P = 0.357), as compared to TP53 wild-type patients. Despite the lack of statistical significance in the METex14 skipping mutation cohort, it is worth mentioning that those with TP53 mutations had comparatively worse prognosis than those without, indicating a clinically significant trend.

In addition to TP53, a study by Schrock et al. showed that other gene amplifications, such as EGFR, frequently occur concomitantly with METex14 alterations (39), which was also observed in our study. In their study, KRAS mutations were observed in 3% of METex14 samples; concurrent MET amplification was identified in 15% of METex14 samples (39). Furthermore, Jamme et al. found that PIK3CA mutations occur in 3% of patients with METex14 mutations (40). Only a small number of published articles have reported concurrent mutations in NSCLC patients with METex14 skipping mutations. In our study, the frequencies of concurrent EGFR amplification, MET amplification, and PIK3CA mutation in NSCLC patients with METex14 skipping mutation were 6.5, 12.9, and 9.7%, respectively, which are comparable with the findings of the studies by Schrock et al. and Jamme et al. (39, 40). Although MET TKIs have demonstrated notable efficacy against advanced NSCLC with MET amplification and METex14 skipping mutation, cases of primary resistance are increasingly observed; the response rate in such cases is lower than that of targeted TKIs of other oncogene-addicted NSCLC.

MET is activated upon binding of HGF, leading to downstream activation of the PI3K and MAPK pathways, subsequently causing tumor proliferation, progression, and metastasis (3–6). The PI3K pathway is frequently dysregulated in NSCLC; PIK3CA is found in 0.1–0.9% of lung cancer cases (41, 42). A study by Jamme et al. suggested that the PIK3CA mutation was commonly found in NSCLC with METex14 skipping mutation; this concurrent mutation plays a role in primary resistance to MET TKIs (40). Several studies have reported the role of PIK3CA alterations in resistance to cancer therapy, including TKI treatment (43, 44). Preclinical data suggest that the PIK3CA mutation E545K decreases the sensitivity of EGFR-mutated cells to EGFR TKIs (45). Regarding patients with EGFR-mutated NSCLC, Eng et al. reported worse OS with a concurrent PIK3CA mutation compared to the absence of this mutation (46).

Our present findings suggest that PIK3CA mutation may be associated with resistance to MET TKIs and is based on the following observations. In our study, 50% of the METex14 skipping mutation cohort and 80% of the MET amplification cohort harboring a PIK3CA mutation showed disease progression with MET TKI therapy, suggesting primary resistance to MET TKIs. In relation to this, five patients, specifically in the MET amplification cohort, with concurrent PIK3CA mutation receiving MET TKI therapy had a PFS of only 1.0 months. This further supported the hypothesis that PIK3CA mutations may be a molecular factor in primary resistance to MET TKIs.

This study has some limitations. Due to its retrospective nature, some clinical information is incomplete, such as the exclusion of several patients due to lack of detailed information and proper follow-up. In addition, not all patient samples were tested using the 168 gene panel; some patients were tested using the 56 gene panel. Therefore, analysis of concurrent molecular factors in patients tested using the 56 gene panel was limited.

After analyzing our data, we speculate the possible association of PIK3CA mutation with primary resistance to MET TKIs in NSCLC patients with MET amplification. Additional studies and experiments must be conducted to elucidate this hypothesis. Moreover, a large prospective cohort study is needed to investigate predictive and prognostic biomarkers for stratifying patients with MET-amplified and METex14-mutated lung cancers.

## Conclusion

In summary, patients with METex14 skipping mutation had a significantly longer PFS than in patients with MET amplification, indicating that MET TKIs could be a better treatment option for patients with the METex14 skipping mutation. Moreover, concurrent mutations may deteriorate the PFS in NSCLC patients with MET amplification and METex14 skipping mutations. TP53 mutations in patients with MET amplification and METex14 skipping mutations were associated with worse survival outcomes than those with the wild type. PIK3CA mutations may confer primary resistance to MET TKIs in patients with MET amplification. These findings contribute to a better understanding of the molecular factors associated with clinical outcomes of NSCLC patients with MET amplification and METex14 alterations.

## Data Availability Statement

The datasets generated for this study are available on request to the corresponding authors.

## Ethics Statement

The studies involving human participants were reviewed and approved by Hunan Cancer Hospital. All patients signed the informed consent form. The patients/participants provided their written informed consent to participate in this study. Written informed consent was obtained from the individual(s) for the publication of any potentially identifiable images or data included in this article.

## Author Contributions

LL and JQ performed the experiment. HY, CZ, YX, and LL provided patient information. SZ collected the data. FK revised the manuscript. JQ and NY were responsible for study conception and design and acquiring financial support. All authors contributed to the article and approved the submitted version.

## Funding

This work was supported by grants from the National Natural Science Foundation of China (No. 81802278) and the Natural Science Foundation of Hunan Province (No.2019JJ50361).

## Conflict of Interest

The authors declare that the research was conducted in the absence of any commercial or financial relationships that could be construed as a potential conflict of interest.

## Supplementary Material

Supplementary Figure 1 NSCLC patients with MET intermediate amplification showed shorter survival outcomes compared to high MET amplification with MET TKI therapy. Kaplan-Meier curves of PFS **(A)** and OS **(B)** of patients treated with MET TKIs in the intermediate and high MET amplification cohorts.

Supplementary Figure 2 NSCLC patients with MET amplification harboring PIK3CA mutations have shorter PFS with MET TKI therapy. Kaplan-Meier curves of PFS **(A)** and OS **(B)** of patients treated with MET TKIs based on presence or absence of concurrent PIK3CA mutation in the MET amplification cohort.

## Abbreviations

NSCLC, non-small cell lung cancer; PFS, progression-free survival; OS, overall survival; EGFR, epidermal growth factor receptor; ALK, anaplastic lymphomakinase; HGF, hepatocyte growth factor; PI3K, phosphatidylinositol-4-bisphophate 3-kinase; MAPK, mitogen-activated protein kinase; ORR, overall response rate; FISH, fluorescence *in situ* hybridization; NGS, next-generation sequencing; CR, complete response; PR, partial response; DCR, disease control rate; SD, stable disease; CNVs, copy number variations.
