# Supplementary figures and images for: Prognosis and Concurrent Genomic Alterations in Patients With Advanced NSCLC Harboring MET Amplification or MET Exon 14 Skipping Mutation Treated With MET Inhibitor: A Retrospective Study
Source: Front Oncol. 2021 Jun 24;11:649766. doi: 10.3389/fonc.2021.649766 (PMC8264054; doi:10.3389/fonc.2021.649766)

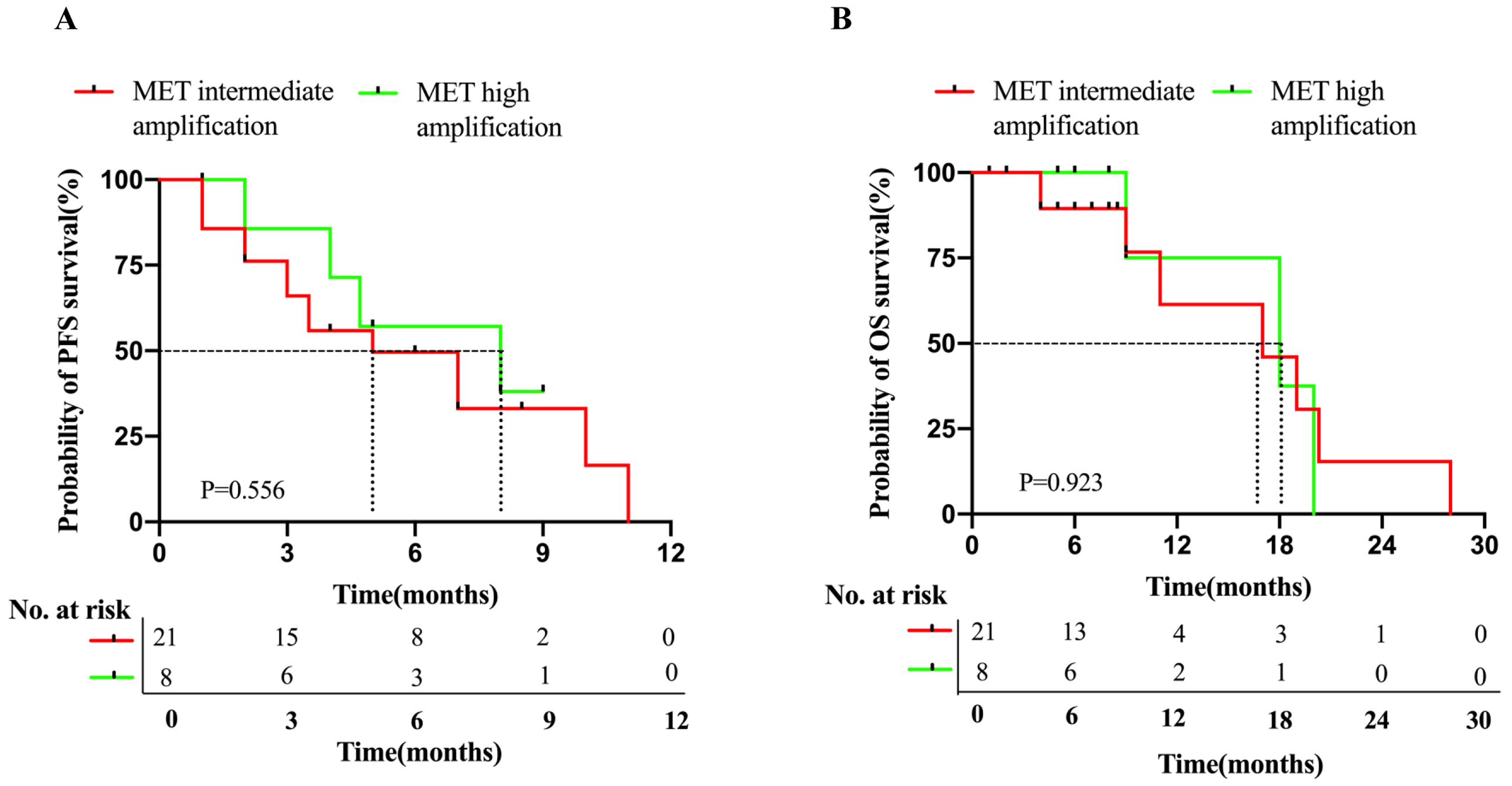

Supplement: Supplementary Figure 1 — NSCLC patients with MET intermediate amplification showed shorter survival outcomes compared to high MET amplification with MET TKI therapy. Kaplan-Meier curves of PFS (A) and OS (B) of patients treated with MET TKIs in the intermediate and high MET amplification cohorts. [file Image_1.tif]

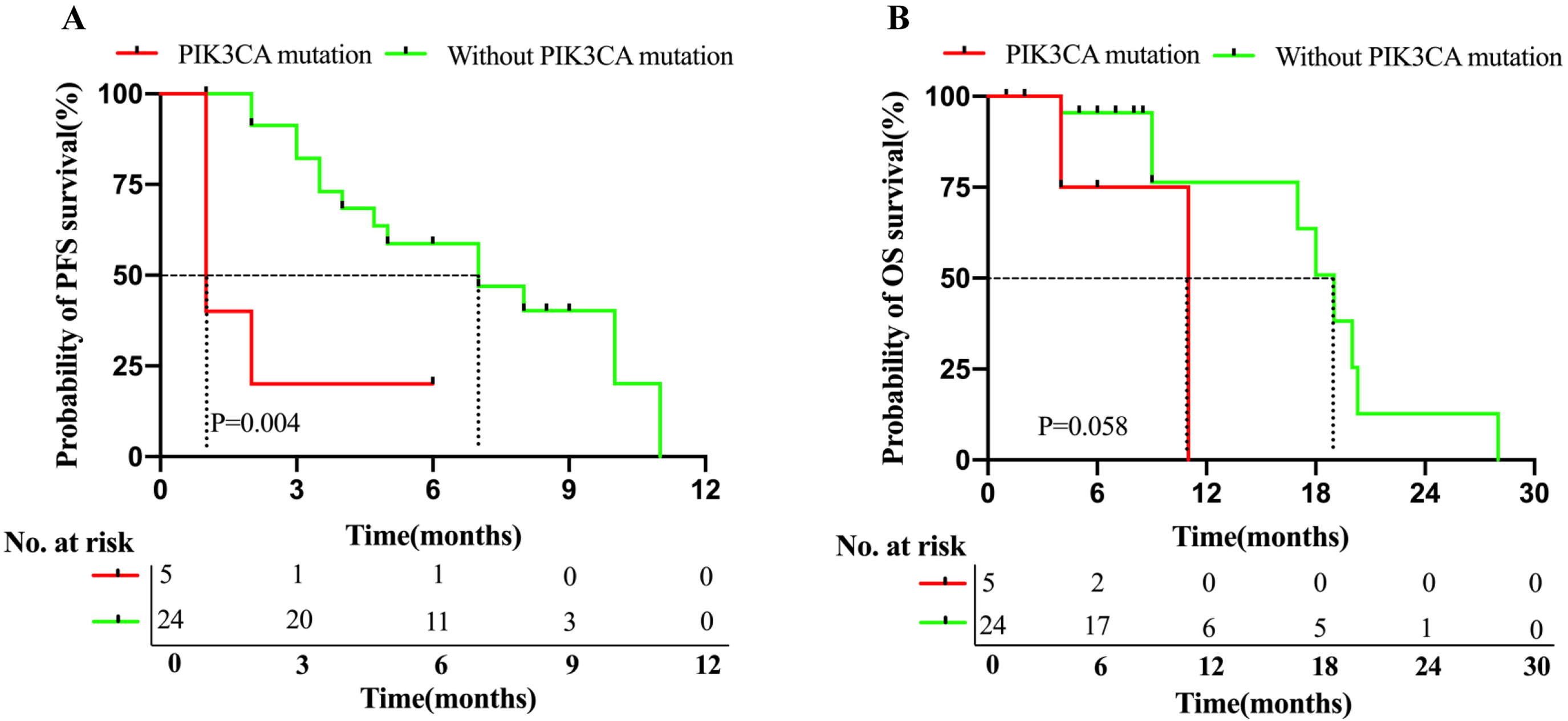

Supplement: Supplementary Figure 2 — NSCLC patients with MET amplification harboring PIK3CA mutations have shorter PFS with MET TKI therapy. Kaplan-Meier curves of PFS (A) and OS (B) of patients treated with MET TKIs based on presence or absence of concurrent PIK3CA mutation in the MET amplification cohort. [file Image_2.tif]
