# Supplementary material for: Prognosis and Concurrent Genomic Alterations in Patients With Advanced NSCLC Harboring MET Amplification or MET Exon 14 Skipping Mutation Treated With MET Inhibitor: A Retrospective Study
Source: Front Oncol. 2021 Jun 24;11:649766. doi: 10.3389/fonc.2021.649766 (PMC8264054; doi:10.3389/fonc.2021.649766)
Supplement: Supplementary file 3 [file DataSheet_1.docx]

Table S1: Summary of MET amplification patients with concurrent mutation

| Patient number | Sample | Concurrent mutation | MET-TKIs | Line of MET-TKIs | Best Response to MET-TKI | PFS(months) | OS(months) |
| --- | --- | --- | --- | --- | --- | --- | --- |
| 1 | Tissue | BRCA CN amp  TP53 mut | Crizotinib | 1 | PR | 4 | 18 |
| 2 | Tissue | TP53 mut  STK-11 mut | Crizotinib | 1 | PR | 5 | 8+ |
| 3 | Tissue | PIK3CA mut  BRCA CN amp | Crizotinib | 1 | PD | 1 | 11 |
| 4 | Tissue | BRCA CN amp  TP53 mut | Crizotinib | 1 | SD | 7 | 9+ |
| 5 | Tissue and blood | PIK3CA mut  BRCA CN amp  TP53 mut  ALK mut | NA | NA | NA | NA | NA |
| 6 | Tissue | BRAF CN amp  TP53 mut | Crizotinib | 1 | SD | 2 | 7+ |
| 7 | Tissue | TP53 mut  STK-11 mut | Crizotinib | 1 | PD | 3.5 | 9 |
| 8 | Tissue | BRAF CN amp | Crizotinib | Subsequent-line | SD | 3 | 4 |
| 9 | Tissue | STK-11 mut  RB1 CN del | Crizotinib | 1 | PR | 9+ | 9+ |
| 10 | Tissue | TP53 mut | Bozitinib | Subsequent-line | SD | 1+ | 1+ |
| 11 | Tissue | NF1 mut | Crizotinib | 1 | PR | 8+ | 8+ |
| 12 | Tissue | RECQL4 mut | NA | NA | NA | NA | NA |
| 13 | Tissue and blood | BRCA CN amp | Crizotinib | 1 | PR | 4+ | 4+ |
| 14 | Tissue | SMARCA4 mut | NA | NA | NA | NA | NA |
| 15 | Tissue | TERT CN amp | NA | NA | NA | NA | NA |
| 16 | Tissue | TP53 mut | NA | NA | NA | NA | NA |
| 17 | Tissue | TP53 mut  PIK3CA mut | Crizotinib | 1 | PD | 1 | 2+ |
| 18 | Tissue | KRAS mut | NA | NA | NA | NA | NA |
| 19 | Tissue | TP53 mut | NA | NA | NA | NA | NA |
| 20 | Tissue | ALK mut | NA | NA | NA | NA | NA |
| 21 | Blood | RB1 CN del  TP53 mut  EGFR CN amp | NA | NA | NA | NA | NA |
| 22 | Tissue | TP53 mut  BRCA CN amp  STK-11 mut  ERBB4 CN amp | Crizotinib | 1 | PR | 3 | 17 |
| 23 | Tissue | TP53 mut  BRCA CN amp | NA | NA | NA | NA | NA |
| 24 | Blood | TP53 mut  PIK3CA mut | Crizotinib | 1 | PR | 6+ | 6+ |
| 25 | Tissue | BRAF CN amp  PIK3CA mut | Crizotinib | 1 | PD | 2 | 4+ |
| 26 | Tissue | TP53 mut  BRCA CN amp  PIK3CA mut | Crizotinib | 1 | PD | 1 | 4 |

Amp: amplification; CN: copy number; Del: deletion; Mut: mutation

Table S2: Summary of METex14 skipping mutation patients with concurrent mutation

| Patient number | Sample | Concurrent mutation | MET-TKIs | Line of MET-TKIs | Best Response to MET-TKI | PFS(months) | OS(months) |
| --- | --- | --- | --- | --- | --- | --- | --- |
| 1 | Tissue | TP53 mut | Crizotinib | 1 | PR | 5 | 12 |
| 2 | Blood | PIK3CA mut | Crizotinib | 1 | PR | 7 | 11 |
| 3 | Tissue | TP53 mut | Crizotinib | 1 | PD | 3 | 4.5 |
| 4 | Tissue | LZTR1 mut | Crizotinib | 1 | PR | 11 | 26 |
| 5 | Tissue | ERBB2 CN amp | Crizotinib | 1 | SD | 5 | 9 |
| 6 | Tissue | RET CN amp | Glumetinib | Subsequent-line | SD | 1+ | 1+ |
| 7 | Tissue | TP53 mut | Crizotinib | 1 | PR | 10+ | 10+ |
| 8 | Blood | CCND3 mut  LPR1B mut | Crizotinib | 1 | SD | 1 | 2.6 |
| 9 | Tissue | MET CN amp | Volitinib | 1 | PR | 8.5 | 14+ |
| 10 | Tissue | MET CN amp  BRAF CN amp | Glumetinib | 1 | PR | 12+ | 12+ |
| 11 | Tissue | CDKN2A mut  TP53 mut | Bozitinib | 1 | PR | 6 | 20 |
| 12 | Tissue | TP53 mut  PIK3CA mut | Glumetinib | 1 | PD | 1 | 7.5 |
| 13 | Tissue | NRAS mut  ERBB2 CN amp | Glumetinib | Subsequent-line | PR | 7+ | 7+ |
| 14 | Tissue | BRAF CN amp  PIK3CA mut | NA | NA | NA | NA | NA |
| 15 | Tissue | EGFR CN amp | Glumetinib | 1 | SD | 3+ | 3+ |
| 16 | Tissue | EGFR CN amp | Crizotinib | 1 | SD | 4 | 6.5 |
| 17 | Tissue | TP53 mut  MET CN amp | Crizotinib | 1 | PR | 11+ | 11+ |
| 18 | Tissue | TP53 mut  MET CN amp | NA | NA | NA | NA | NA |
| 19 | Blood | BRAF CN amp | Crizotinib | Subsequent-line | PR | 12 | 26 |
| 20 | Tissue | BRAF CN amp | Volitinib | 1 | SD | 4 | 9 |

Amp: amplification; CN: copy number; Del: deletion;Mut: mutation
